# Functional Genetic Variants in *ATG10* Are Associated with Acute Myeloid Leukemia

**DOI:** 10.3390/cancers13061344

**Published:** 2021-03-16

**Authors:** Isabel Castro, Belém Sampaio-Marques, Anabela C. Areias, Hugo Sousa, Ângela Fernandes, José Manuel Sanchez-Maldonado, Cristina Cunha, Agostinho Carvalho, Juan Sainz, Paula Ludovico

**Affiliations:** 1 Life and Health Sciences Research Institute (ICVS), School of Medicine, University of Minho, 4710-057 Braga, Portugal; isabel.m.s.castro@gmail.com (I.C.); mbmarques@med.uminho.pt (B.S.-M.); anabelac.areias@gmail.com (A.C.A.); hugomls@gmail.com (H.S.); be.amafernandes@gmail.com (Â.F.); cristinacunha@med.uminho.pt (C.C.); agostinhocarvalho@med.uminho.pt (A.C.); 2 ICVS/3B’s-PT Government Associate Laboratory, 4806-909 Braga/Guimarães, Portugal; 3 Virology Service and Molecular Oncology and Viral Pathology Group (CI-IPOP), Portuguese Oncology Institute of Porto (IPO Porto), 4200-072 Porto, Portugal; 4 Institute for Research and Innovation in Health (i3S), University of Porto, 4200-135 Porto, Portugal; 5 Genomic Oncology Area, GENYO, Centre for Genomics and Oncological Research, Pfizer/University of Granada/Andalusian Regional Government, PTS, Avda. de la Ilustración, 114, 18016 Granada, Spain; josemanuel.sanchez@genyo.es (J.M.S.-M.); juan.sainz@genyo.es (J.S.); 6 Hematology Department, Virgen de las Nieves University Hospital, Avda. Fuerzas Armadas s/n, 18012 Granada, Spain; 7 Instituto de Investigación Biosanataria (IBs. Granada), 18012 Granada, Spain; 8 Department of Medicine, University of Granada, Avda. De la Investigación, 11, 18016 Granada, Spain

**Keywords:** acute myeloid leukemia, *ATG10*, autophagy, single nucleotide polymorphism

## Abstract

**Simple Summary:**

Acute myeloid leukemia (AML) is a hematological neoplasm with a very poor survival rate. To date, diagnostic tools to monitor individuals at higher risk of developing AML are scarce. Single nucleotide polymorphisms (SNPs) have emerged as good candidates for disease prevention. AML is characterized by altered autophagy, a vital mechanism to remove and recycle unnecessary or dysfunctional cellular components. *ATG10* is one of the autophagy core genes involved in the autophagosome formation. We hypothesize that SNPs located in regulatory regions of the *ATG10* gene could predispose individuals to AML development. We therefore genotyped three SNPs within the *ATG10* locus. We identified the *ATG10*_rs3734114_ as a potential risk factor for developing AML, whereas the *ATG10*_rs1864182_ was associated with decreased risk. These findings highlight *ATG10* as a key regulator of susceptibility to AML. Furthermore, we believe that *ATG10* SNPs could be exploited in the clinical setting as an AML prevention strategy.

**Abstract:**

Acute myeloid leukemia (AML) is the most common acute leukemia, characterized by a heterogeneous genetic landscape contributing, among others, to the occurrence of metabolic reprogramming. Autophagy, a key player on metabolism, plays an essential role in AML. Here, we examined the association of three potentially functional genetic polymorphisms in the *ATG10* gene, central for the autophagosome formation. We screened a multicenter cohort involving 309 AML patients and 356 healthy subjects for three *ATG10* SNPs: rs1864182T>G, rs1864183C>T and rs3734114T>C. The functional consequences of the *ATG10* SNPs in its canonical function were investigated in vitro using peripheral blood mononuclear cells from a cohort of 46 healthy individuals. Logistic regression analysis adjusted for age and gender revealed that patients carrying the *ATG10*_rs1864182G_ allele showed a significantly decreased risk of developing AML (OR [odds ratio] = 0.58, *p* = 0.001), whereas patients carrying the homozygous *ATG10*_rs3734114C_ allele had a significantly increased risk of developing AML (OR = 2.70, *p* = 0.004). Functional analysis showed that individuals carrying the *ATG10*_rs1864182G_ allele had decreased autophagy when compared to homozygous major allele carriers. Our results uncover the potential of screening for *ATG10* genetic variants in AML prevention strategies, in particular for subjects carrying other AML risk factors such as elderly individuals with clonal hematopoiesis of indeterminate potential.

## 1. Introduction

Acute myeloid leukemia (AML) is the most common acute leukemia in adults with an average age of disease diagnosis above 65 years old [1]. It is a highly heterogeneous clonal disorder characterized by an impairment in myeloid cellular differentiation and deregulated proliferation, leading to an accumulation of immature myeloid progenitor cells in the bone marrow (BM), peripheral blood, and other tissues. Patients undergoing chemotherapy, radiation, or with a genetic predisposition to myeloid neoplasms are at a higher risk of developing AML [2]. Furthermore, it has been reported that individuals presenting an age-related condition named clonal hematopoiesis of indeterminate potential (CHIP), are also at higher risk of developing AML. CHIP is characterized by the expansion of hematopoietic stem cell (HSC) clones, harboring specific, disruptive, and recurrent somatic mutations [3]. Thus, searching for markers of AML predisposition and progression is of important clinical relevance.

Single nucleotide polymorphisms (SNPs) have emerged as new predictors of diseases like cancer and as indicators of effectiveness of chemotherapy response. To date, the majority of SNPs reported in association with AML are focused on the response to oncological therapies [4,5,6,7]. For example, the *STAT3*_rs9909659_ could be used to predict the treatment outcome to chemotherapy with daunorubicin and cytarabine [8]. Moreover, a SNP located in the mutational hotspot of Wilms tumor 1 (*WT1*) was identified as a novel favorable prognostic marker in cytogenetically normal AML [9]. The SNP rs11554137 present at the isocitrate dehydrogenase 1 (*IDH1*) gene, a citric acid cycle enzyme involved in metabolism, was shown to be a negative outcome predictor of AML [10,11]. The presence of *IDH1*_rs11554137_ results in the production of the oncometabolite (R)-2-hydroxyglutarate leading to the poor prognosis of normal karyotype adult AML. This particular finding may indicate the potential presence of other SNPs involved in the complex pathways of metabolism and perhaps in cellular recycling mechanisms such as autophagy. Interestingly, AML is characterized by changes in autophagy flux in both pre-leukemic cells as well as in leukemic stem cells, as a consequence of acquired somatic mutations, a process associated with increased age [12]. Despite these advances, the need for finding novel SNPs associated with AML predisposition remains. Only few SNPs have been associated with higher risk of developing myeloid leukemia [13]. As an example, the presence of the B-cell lymphoma 2 (*BCL2*) polymorphism, *BCL2*_938C/A_ and the *BCL2*-associated X protein (*BAX*) polymorphism, *BAX*_248GG_ were significantly associated with an increased risk of AML occurrence [14]. Furthermore, two SNPs in *CASP9* gene (rs1263 and rs712) are associated with AML susceptibility [15].

Autophagy is a coordinated process responsible for the removal of misfolded proteins and dysfunctional organelles from cells by means of lysosomal degradation [16]. Autophagy plays a key role in cancer since it can either suppress tumorigenesis by inhibiting cancer cell survival, or facilitate tumorigenesis by promoting cancer cell proliferation and tumor growth [17,18]. Recent studies have shown autophagy-related prognostic signature for AML prediction [19]. An important step in autophagy is the autophagosome formation, which is mediated by a set of ATG proteins such as *ATG10*. *ATG10* is an E2-like enzyme that catalyzes the conjugation reaction between ATG12 and ATG5, when interacting with ATG7, an essential set for autophagy vesicle formation [20]. Interestingly, apart from *ATG10*’s role in autophagy, *ATG10* seems to have a non-canonical function in inflammation [21,22].

The Human Genome Project has identified many *ATG10* SNPs, but to date only few were investigated in AML research [23]. The two missense *ATG10*_rs1864182_ and *ATG10*_rs1864183_ were shown to be situated in the region containing enhancer histone markers in induced pluripotent stem cells (iPSCs), and they are possible motifs altering binding of the transcription factors DMRT1 and Myc [24]. Curiously, DMRT1 and Myc are key players in the development of AML [25,26]. Another missense *ATG10* SNP, the *ATG10*_rs3734114_, was described for its putative involvement in lung, thyroid, brain and bladder cancers [27,28,29,30]. For these reasons, in this work we studied the association of AML development with these three *ATG10* SNPs (*ATG10*_rs1864182_, *ATG10*_rs1864183_ and *ATG10*_rs3734114_), using a Spanish cohort of AML patients and healthy donors, with a total of 665 participants. We further evaluated the autophagic function of the associated SNPs using an independent cohort of 46 healthy donors. Our results demonstrate the importance of autophagy-related *ATG10* SNPs in AML development.

## 2. Materials and Methods

### 2.1. Ethics Statement

Two independent cohorts were used for this study. Cohort 1 consisted of 665 Spanish subjects, including 309 AML subjects and 356 healthy subjects that were ascertained through the NuCLEAR consortium [31]. All ethical issues concerning this cohort are described in [31]. Cohort 2 included 46 healthy Portuguese subjects. The study was approved by the Ethics Committee for Research in Life and Health Sciences (CEICVS) at University of Minho (SECVS 010/2015).

### 2.2. DNA Extraction, SNP Selection Criteria and Genotyping

Genomic DNA from whole blood samples was isolated using the NZY Blood gDNA Isolation kit (NZYTech, Lisbon, Portugal) according to the manufacturer’s instructions. The frequency of each selected SNP in the Caucasian population was considered, based on the International HapMap Project (HapMap-CEU). Genotyping of the *ATG10*_rs1864182_ and *ATG10*_rs1864183_ was performed using the KASPar genotyping chemistry (LGC Genomics, Hoddesdon, UK) and *ATG10*_rs3734114_ using TaqMan SNP Genotyping Assay (Thermo Fisher Scientific, MA, USA), following the manufacturer’s instructions. Call rate for all tested SNPs was >98%. Quality control for the genotyping results were achieved with negative controls and randomly selected samples included as duplicates.

### 2.3. Association Studies

For both cohorts, contingency tables were calculated. Association studies were performed following the methods described in [32] using Rstudio version 1.4.1103. The major allele was considered the most frequent allele in the European population based on National Library of Medicine (NIH). Hardy–Weinberg Equilibrium (HWE) and corresponding significant differences were calculated for the control population of each SNP using a standard observed-expected chi-square (χ^2^) test. The genotypic odds ratios (OR) for the dominant and recessive model as well as the corresponding confidence intervals were calculated, and both χ^2^ and Fischer exact (FE) test were performed. Logistic regression for the dominant and recessive model adjusted for age and gender was performed to control for these possible confounding factors. Since we simultaneously assessed other autophagy SNPs in this study, we performed a correction for multiple testing using the Bonferroni method (Supplementary Table S1). Linkage Disequilibrium (LD) between the three studied *ATG10* SNPs studied was analyzed using Haploview software.

### 2.4. mRNA Expression Analysis by qRT-PCR

Analysis of quantitative mRNA expression was accomplished according to MIQE guidelines [33]. In brief, *ATG10* mRNA transcripts expression levels were measured by quantitative real-time PCR (qPCR) (Supplementary Table S2). The expression levels of these transcripts were normalized against that of three reference genes: β-2-microglobulin (*B2M*), ribosomal protein L13a (*RPL13A*), Glyceraldehyde 3-phosphate dehydrogenase (*GAPDH*) (Supplementary Table S2).

The RNA extraction was performed using the NZYol RNA Isolation Reagent (NZYTech). Total RNA (250 ng) was reverse-transcribed into cDNA in a 20 μL reaction mixture using the NZY First-Strand cDNA Synthesis kit (NZYTech). Then, in a 20 μL reaction mixture, 50 ng of cDNA, of each sample, were tested in duplicate in a 96-well plate (Bio-Rad, CA, USA). The qPCR was processed in a CFX96™ Real Time System (Bio-Rad), with the NZY qPCR Green Master Mix (NZYTech), according to the manufacturer’s instructions. A blank (no-template control) was also included in each assay run. The qPCR conditions consisted of one hold at 95 °C for 1 min, followed by 45 cycles of 15 min at 95 °C, 20 s at 60 °C and 20 s at 72 °C. At the end, a melting-curve was acquired to evaluate the PCR specificity, contamination and the absence of primer dimers. Furthermore, the PCR efficiency was also tested according to methods described in [33]. Final values of relative expression levels of *ATG10* mRNA transcripts were determined by correcting for the differences in efficiencies between target and reference genes using the gene expression software of the CFX manager program (Bio-Rad).

### 2.5. Protein Expression and Immunoblot Analysis

For protein extraction, 50 µL of lysis buffer was used, containing 1% NP-40, 500 mM Tris HCL, 2.5 M NaCl, 20 mM EDTA, Phosphatase and Protease inhibitors (Roche, Mannheim, Germany), at pH 7.2, followed by a sonication process. For the immunoblotting assay, 20 µg of total protein extract were resolved in a 12% SDS gel and transferred to a nitrocellulose membrane during 10 min, using the Trans-Blot Turbo transfer system (Bio-Rad). Then, membranes were blocked using Tris-buffered saline (TBS) with 0.1% tween 20 (TBS-T) plus 5% bovine serum albumin (BSA) and incubated overnight at 4 °C. This incubation was performed with the polyclonal primary antibodies resuspended in 1% BSA: Rabbit anti-LC3A/B Antibody (1:1000, Cell-Signaling, MA, USA), mouse anti-p62 (1:1000, Abcam, Cambridge, UK), anti-mono and polyubiquitination conjugated (1:1000, Enzo Biochem, NY, USA ) or mouse anti-alpha-actin (1:1000, Millipore, MA, USA), followed by one hour incubation with the secondary antibodies (HRP, anti-rabbit, anti-mouse 1:5000) (Bio-Rad). Blots were developed with the SuperSignal West Femto Maximum Sensitivity Substrate (Thermo Fisher Scientific) or the Clarity Western ECL Substrate (Bio-Rad). Digital images were obtained in a ChemiDoc XRS System (Bio-Rad) and the densitometry analysis of the bands was performed with the Quantity One software V4.6.5 (Bio-Rad).

### 2.6. Peripheral Blood Mononuclear Cells (PBMC) Isolation

Venous blood was drawn into 10 mL EDTA tubes. Blood was diluted in PBS (1:1) and fractions were separated by Histopaque^®^-1077 (Sigma-Aldrich, MO, USA) density gradient centrifugation according to the protocol of the manufacturer. PBMCs were washed twice with PBS and resuspended in RPMI-1640 medium (Gibco, Paisley, UK) supplemented 10% FBS (Gibco) and antibiotic-antimycotic solution (Sigma-Aldrich).

### 2.7. Autophagy Flux Analysis

Briefly, PBMCs were plated in 12-well round-bottom plates (Corning, NY, USA) at a concentration of 5 × 10^5^ cell/mL and incubated, with or without 10 µM of metformin (Sigma-Aldrich), for 4 h. Furthermore, in order to block the autophagy flux and to allow the accumulation of LC3-II [34], 2 h before the end of the treatment, in each condition, cells were also incubated with 10 nM of bafilomycin (Baf A1) (Sigma-Aldrich). After incubation, cells were lysed and immunoblotting assays were performed for autophagy-related proteins, as described in 2.5. To quantify autophagy synthesis, the ratio of the values of the cells treated with metformin and Baf A1 against those for condition without metformin but with Baf A1 treatment was determined. To quantify autophagy degradation, the ratio between the densitometric values of cells treated with metformin in the presence or absence of Baf A1 was determined, according to autophagy standard guidelines [34]. Due to the number of samples in our study and the limited amount of protein per sample, technical replicates of the blots were not performed, however most conditions contain enough samples to warrant a reliable statistical analysis, with exception of samples for *ATG10*_rs3734114C/C_ for which statistical tests were not performed.

### 2.8. Statistical Analysis

Each condition was tested for normality before statistical analysis using a Shapiro-Wilk test. To compare one or two normally distributed samples a *t*-test was performed. For more than two samples an ANOVA test was performed. When at least one of the samples was non-normally distributed, the Kruskal–Wallis test was performed. All statistical procedures were conducted using GraphPad Prism.

## 3. Results

### 3.1. Demographic Characterization of the Cohorts

Two European cohorts were used, one for SNP analysis (cohort 1) and the other for functional studies (cohort 2). Demographic characterization of the cohorts is detailed in Table 1. Cohort 1 consisted of 665 Spanish subjects from the NuCLEAR consortium [31] comprising 309 AML patients and 356 healthy subjects. Gender balance was observed in cohort 1 (χ^2^ test, *p* = 0.612), while age distribution was statistically different between AML cases and healthy controls (56 ± 6 and 58 ± 17 years mean age, respectively, *p* < 0.05). The association analyses were adjusted for age and gender. Cohort 2, which included 46 healthy subjects, was used for functional studies (Table 1).

### 3.2. Linkage Analysis of *ATG10* SNPs

The *ATG10* polymorphisms studied are located on the chromosome 5, at the positions 82253421, 82253397 and 82058570 for the *ATG10*_rs1864182_, *ATG10*_rs1864183_ and *ATG10*_rs3734114_, respectively (Table 2, Supplementary Figure S1A). Random association of the SNPs alleles in the *ATG10* locus was analyzed by calculating the allelic linkage (Supplementary Figure S1B). Linkage disequilibrium (LD) analysis showed a close association of *ATG10*_rs1864182_ with *ATG10*_rs1864183_ (coefficient of LD (D’) of 0.93 and r^2^ of 0.62). A moderate linkage between these two SNPs was expected due to their loci proximity. For the linkage between *ATG10*_rs3734114_ and *ATG10*_rs1864183_ or *ATG10*_rs1864182_, D’ and r^2^ values were low, ranging from 0.17–0.26 and 0.0–0.02 respectively, showing that the *ATG10*_rs3734114_ represents an independent signal.

### 3.3. Associations of Genetic Variants on *ATG10* with AML

Associations of *ATG10*_rs1864182_, *ATG10*_rs1864183_ and *ATG10*_rs3734114_ with AML were studied in cohort 1. Minor allele frequencies in the control population, as well as the nucleotide changes for each SNP are presented in Table 2 and Supplementary Figure S1A, respectively. All SNPs were in Hardy–Weinberg equilibrium (HWE) (Table 2).

Allele and genotype frequencies were in line with those reported by the NIH for the CEU population (https://www.ncbi.nlm.nih.gov/snp/, accessed 12 January 2021) (Table 2 and Table 3). The genotype distributions for the *ATG10*_rs3734114_ were different between patients and controls (χ^2^ test, *p_adjusted_* = 0.012, respectively) (Table 3). Logistic regression analysis adjusted for age and gender revealed that AML patients carrying the *ATG10*_rs1864182G_ allele showed a significant decreased risk of developing AML (OR_Dominant_ = 0.58, with a 95% confidence interval [CI] = 0.42–0.80, Table 3), whereas patients carrying the *ATG10*_rs3734114C/C_ genotype had a significant increased risk of developing AML when compared with those carrying the most common allele (OR_Recessive_ = 2.70, 95% CI = 1.36–5.34, Table 3). All results were corrected for multiple testing using Bonferroni correction (Supplementary Table S1). Our analysis confirmed that older subjects [35,36] and males [37] are at higher risk of developing AML. In summary, our results proposed a decreased risk of developing AML when carrying the *ATG10*_rs1864182G_, whereas identified *ATG10*_rs3734114C/C_ as a risk factor for the development of AML. 

### 3.4. Impact of *ATG10*_rs1864182_ and *ATG10*_rs3734114_ in mRNA and Protein Levels

The impact of *ATG10* SNPs associated with AML on mRNA expression and on the protein levels of *ATG10* isoforms was assessed in PBMCs of healthy donors from cohort 2. The genotype characterization of *ATG10* SNPs in cohort 2 is presented in Table 4. The similarity in genotype frequencies between cohort 2 and the CEU population (https://www.ncbi.nlm.nih.gov/snp/, accessed 12 January 2021) supported the use of this cohort. For the *ATG10*_rs1864182_, we followed the predicted dominant model as indicated by our analysis (Table 3) and therefore, TG and GG genotypes were combined in one group. Regarding *ATG10*_rs3734114_, the predictive model suggested a recessive behavior of the alternative C allele and therefore both TT and TC genotypes were combined. However, due to the low frequency of CC (4.3% in cohort 2), statistical analysis was not conducted for this SNP. 

**Figure 1:**
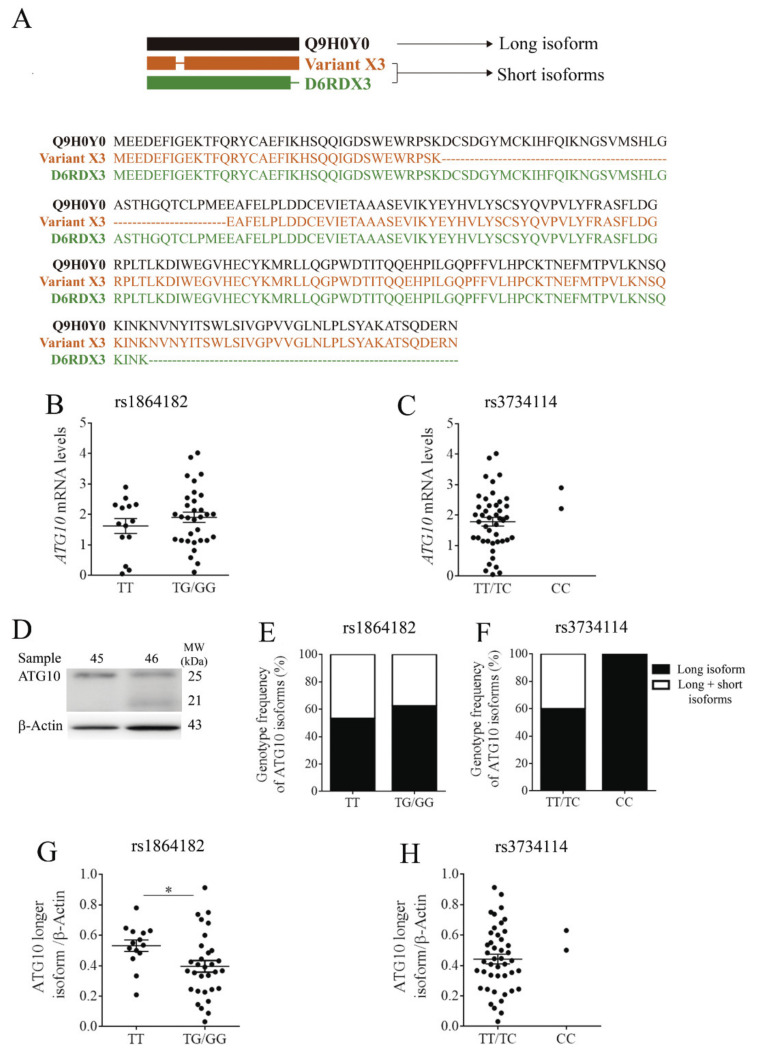
(**A**) Schematic representation of *ATG10* protein sequence and the corresponding long (Q9H0Y0, black) and short (Variant X3 and D6RDX3, orange and green, respectively) isoforms. (**B**,**C**) *ATG10* mRNA levels of individuals carrying *ATG10*_rs1864182_ and *ATG10*_rs374114_, respectively. (**D**) Illustrative western blot analysis of *ATG10* isoforms: long (25kDa, sample 45) and short (21kDa, sample 46). (**E**,**F**) Genotype frequencies of short and long *ATG10* isoforms in the dominant model for rs1864182 and recessive model for rs374114, respectively. Black squares represent the percentage of individuals with the presence of the long isoform, whereas white squares correspond to the percentage of individuals with long and short isoforms. (**G**) Levels of *ATG10* long isoform for the *ATG10*_rs1864182_ carriers (* denotes *p* < 0.05, unpaired *t*-test). (**H**) *ATG10* long isoform levels for the *ATG10*_rs3734114_ carriers. Protein levels were normalized by β-actin level. Error bars denote one standard deviation.

As mentioned earlier, *ATG10* is an E2-like enzyme involved in E2 ubiquitin-like modifications, crucial for autophagosome formation [38]. Previous studies showed the existence of at least three different *ATG10* protein isoforms, Q9H0Y0 (the longest isoform) and variants X3 and D6RDX3 (shorter isoforms) (Figure 1A) [22]. Q9H0Y0 is composed of 663 nucleotides, encoding 220 amino acids, whereas the isoform Variant X3 has 555 nucleotides coding for 184 amino acids (from www.ncbi.nlm.nih.gov/CCDS/, accessed 12 January 2021). Both short isoforms present identical sequences with the exception of a deletion of 36 amino acids in the N-terminal of Variant X3 [21,22]. The D6RDX3 isoform also has 555 nucleotides coding for 184 amino acids, but the 36 amino acids deletion occur at the C-terminal region. As a result of these deletions, isoforms Variant X3 and D6RDX3 have the same molecular weight of 21 kDa, whereas the long isoform has a molecular weight of 25 kDa.

Despite the *ATG10* isoforms described, the study of the *ATG10* mRNA levels was performed using a set of primers allowing for the amplification of a region common to all isoforms (Supplementary Table S2). The results demonstrated that the presence of the *ATG10*_rs1864182_ did not have any significant impact on *ATG10* mRNA levels (Figure 1B). With regards to the presence of *ATG10*_rs3734114_, the two recessive individuals for the alternative allele C had a higher mRNA expression than heterozygous individuals, although the significance of this trend could not be verified by statistical analysis (Figure 1C).

The selected *ATG10* antibody allowed distinguishing the longest from the shorter isoforms (Figure 1D). Immunoblot analysis revealed that none of the *ATG10* isoforms are predominantly associated with a particular *ATG10*_rs1864182_ genotype (Figure 1E). No conclusions could be drawn for *ATG10*_rs3734114_, but it is nonetheless interesting to note that the two individuals carrying the homozygous alternative allele C only displayed the longest *ATG10* isoform (Figure 1F).

We next evaluated the protein levels of the *ATG10* long isoform, which is mainly involved in autophagy catalytic function [21,22]. In the presence of the *ATG10*_rs1864182G_, a statistically significant decrease of 25% in the *ATG10* long isoform levels was observed (Figure 1G). It is interesting to note that both healthy donors with homozygous high-risk *ATG10*_rs3734114_ genotype showed a higher mean value of *ATG10* long isoform levels than when in the presence of major allele (Figure 1H). These results are in accordance with the higher mRNA expression levels observed (Figure 1C). Based on these results, we further evaluated the functional impact of these *ATG10* SNPs on *ATG10* canonical function in autophagy.

### 3.5. Functional Effects of *ATG10*_rs1864182_ and *ATG10*_rs3734114_ on Autophagy

**Figure 2:**
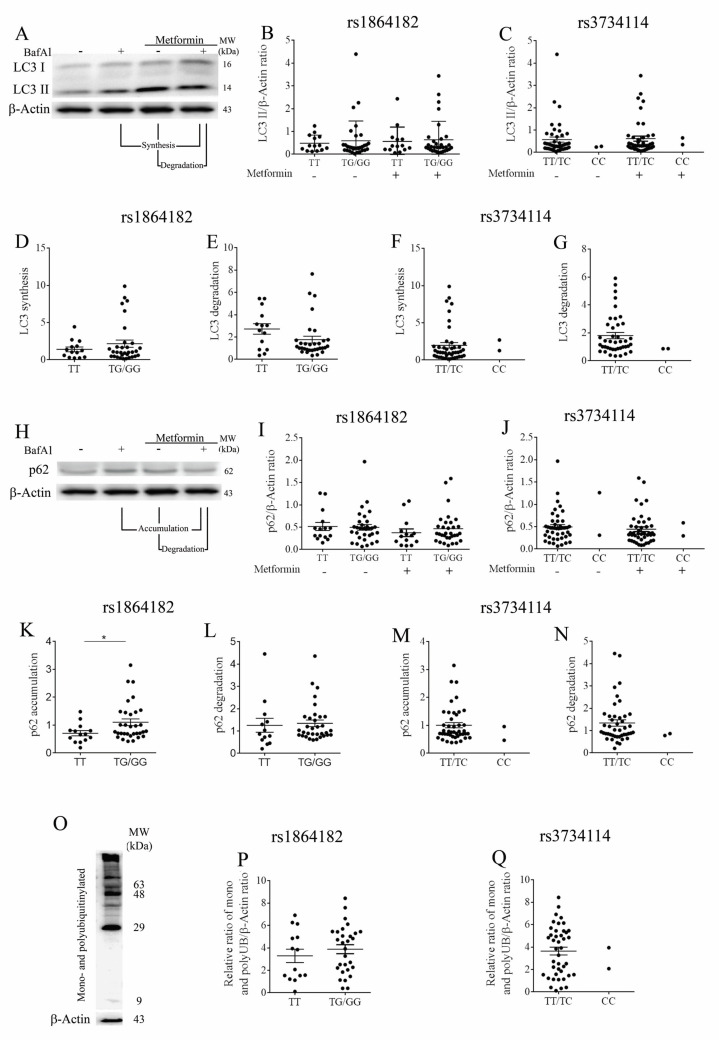
(**A**) Representative blot for study of LC3 processing. For *ATG10*_rs1864182_ and *ATG10*_rs3734114_: (**B**,**C**) LC3-II levels; (**D**,**F**) LC3 synthesis and (**E**,**G**) degradation. (**H**) Representative blot for p62 levels. For *ATG10*_rs1864182_ and *ATG10*_rs3734114_: (**I**,**J**) p62 levels; (**K**,**M**) p62 accumulation and (**L**,**N**) degradation. (**O**) Representative blot for ubiquitination profile and (**P**,**Q**) graphical representation of the intensity of total UB/β-actin obtained by densitometric analysis, for *ATG10*_rs1864182_ and *ATG10*_rs3734114_, respectively. Statistical significance of the data was determined by Mann Whitney test (* *p* ≤ 0.05). The error bars represent one standard error of the mean (SEM).

To investigate whether autophagy is affected by the presence of the protective or risk in *ATG10* SNPs identified in our association analysis, we evaluated autophagic flux in PBMCs from healthy donors. Measurements were performed in both basal and stimulated conditions. For the latter, cells were treated with the autophagy inducer metformin during 4 h [39]. Autophagy flux was assessed by LC3 processing performed according to gold standard guidelines [34]. During autophagy, LC3-I is conjugated to phosphatidylethanolamine (PE) to form LC3-II, which co-localizes at the autophagosome membranes thus reflecting the number of autophagosomes and autophagy-related structures. When analyzing LC3-II levels in PBMCs of healthy donors (Figure 2A) carrying either *ATG10*_rs1864182_ or *ATG10*_rs3734114_, no significant differences were found in both basal and stimulated conditions between genotypes (Figure 2B,C). LC3-II has a high recycling turnover, synthesis and degradation. Therefore, we used an autophagosome-lysosome fusion inhibitor, bafilomycin A1 (Baf A1), to block this process, allowing the accumulation of autophagosomes. Under normal autophagic flux an accumulation of LC3-II is expected, and alterations in LC3-II accumulation suggest an imbalance by defaulted synthesis and/or degradation. LC3-II synthesis was assessed by computing the ratio of the LC3-II protein levels from stimulated conditions (treated with metformin) plus Baf A1 over the LC3-II protein levels of the samples under basal conditions with Baf A1 alone. The LC3-II degradation was also assessed by computing the ratio of the LC3-II protein levels in stimulated conditions plus Baf A1 over the LC3-II protein levels obtained under stimulated conditions only (Figure 2A) [34].

For the *ATG10*_rs1864182_, LC3-II synthesis was not significantly affected by the presence of the alternative allele (Figure 2D). On the other hand, the LC3-II degradation was reduced in individuals with this SNP, although this trend did not achieve statistical significance (Figure 2E). These observations suggested that the *ATG10*_rs1864182G_ may be associated with a decreased autophagy flux. No differences were observed when evaluating LC3-II synthesis and degradation in individuals with and without the *ATG10*_rs374114C/C_. However, note the sample size of *n* = 2 in this case (Figure 2F,G).

p62 is a multifunctional protein involved in different pathways, including autophagy and the proteasomal degradation of ubiquitinated proteins. p62 is an autophagy substrate and like LC3-II has been extensively used as a reporter of autophagy activity [40]. Alterations in p62 accumulation and degradation also suggest changes in autophagic flux. Indeed, LC3 co-localizes and is immunoprecipitated with p62, suggesting that these two proteins are involved in the same complexes [41]. Furthermore, it is described that the turnover of p62 occurs in the same conditions as LC3 [41]. Altogether, autophagy inhibition leads to an increase of p62 protein levels. We next examined the accumulation and the degradation of p62 (Figure 2H). The total p62 levels for both of the studied SNPs did not reveal significant differences when comparing individuals with the presence of minor alleles (Figure 2I,J). However, when analyzing p62 accumulation and degradation, as done for LC3-II [41], in the presence of *ATG10*_rs1864182G_, we found a significant increase in the p62 accumulation (Figure 2K), without major alterations on p62 degradation (Figure 2L). Decrease of LC3-II degradation and an increase of p62 accumulation is associated with a reduction of autophagy flux [34]. No differences in p62 were observed between the two groups with *ATG10*_rs374114C/C_ (Figure 2M,N, note *n* = 2).

Impaired autophagy leads to an accumulation of ubiquitinated proteins [42], which could be associated with a faulty degradative process such as the ubiquitin proteasome system (UPS). Ubiquitination is a well-known post-translational modification, involving the conjugation of different ubiquitin length chains to proteins [43]. Depending on the distinct structure of the ubiquitin chains, the protein outcome will be different (degradation, signal transduction or subcellular localization) [44]. Thus, to further support the defective autophagy observed in individuals presenting the *ATG10*_rs1864182G_ and to evaluate its impact on the proteasomal degradation, we evaluated the total ubiquitination profile by immunoblot analysis (Figure 2O). Our results revealed that individuals carrying the *ATG10*_rs1864182G_ tended to present increased levels of ubiquitinated proteins (Figure 2P). Regarding the *ATG10*_rs374114C/C_ no major conclusions could be drawn (Figure 2Q).

In summary, concerning the *ATG10*_rs1864182_, the results herein suggest a diminished autophagic flux in individuals with the protective G allele, resulting from decreased LC3-II degradation and consequent p62 accumulation. This also suggests that individuals who do not display the protective allele exhibit higher autophagy activity. Furthermore, these changes in autophagy appear to have a mild impact on the UPS system, since individuals presenting the *ATG10*_rs1864182G_ appear to have increased levels of ubiquitinated proteins. For the *ATG10*_rs374114_, no major conclusions could be drawn, due to the low frequency of the risk homozygous allele in the population.

## 4. Discussion

AML is a severe disease with a rapid progression and a high fatality rate, particularly in the elderly. AML affects 3 to 4 persons per 100,000 per year (https://seer.cancer.gov/statfacts/html/amyl.html, accessed 12 January 2021). The existence of a familiar or personal medical history (e.g., close relatives with AML, blood disorders, and genetic syndromes), the incidence of a primary cancer undergoing chemotherapy, and the presence of clonal hematopoiesis of indeterminate potential (CHIP) are well-known risk factors for AML development. Therefore, the development of new strategies for risk stratification of individuals at higher risk will be important to place them on the medical radar.

After studying the impact of different SNPs on autophagy and other-related processes (Supplementary Table S1), in this study, we evaluated the association between three SNPs in the *ATG10* gene with AML in a case-control study. SNPs have been recognized as risk factors for disease development and are an excellent tool to investigate etiology, inter-individual differences in treatment response, and outcomes of cancers [45]. Genetic variations of autophagy core genes have been the focus of research in several human cancers [46]. The *ATG10*_rs1864182_ has been previously associated with decreased risk of breast cancer [47] and melanoma [48]. However, other studies have shown the association of the *ATG10*_rs1864182_ with poor lung cancer survival in particular on the non-small cell lung cancer (NSCLC) [24]. To the best of our knowledge, our study is the first to examine the association of *ATG10* SNPs with AML and to explore the functional implications of these SNPs on autophagy. We described the association of *ATG10*_rs1864182G_ with a decreased probability of developing AML. *ATG10*_rs1864182_ was also shown to serve as biomarker for primary or acquired resistance to chemotherapy when using gefitinib (an epidermal growth factor receptor (EGFR)-TKI drug) in advanced lung adenocarcinoma patients with EGFR mutations [49]. Studying the involvement of *ATG10*_rs1864182_ in the resistance to chemotherapy may be a useful future biomarker for tailoring AML treatments.

*ATG10*_rs3734114_ was recently associated with increased brain metastasis in NSCLC patients [30]. The study showed that patients carrying the *ATG10*_rs3734114CT/CC_ genotypes had an increased cumulative brain metastasis hazard of 46% compared to 13% in patients with the TT genotype [30]. In our studies, we found for the first time an association of the *ATG10*_rs3734114C/C_ with a higher risk of developing AML, suggesting that the *ATG10*_rs3734114C/C_ variant could be a risk factor for AML.

Finally, in our study, we did not find any association between the *ATG10*_rs1864183_ and AML. Nevertheless, its association with the development of pharyngeal cancer has been previously described [50]. Altogether, our results show the potential application of the *ATG10*_rs1864182_ and *ATG10*_rs3734114_ as risk biomarkers for AML.

Random association between *ATG10*_rs1864182_ and *ATG10*_rs3734114_ was observed when analyzing the LD between the two alleles. This indicates that the SNPs are not mutually exclusive, even though they have opposite association with AML. Because of the low probability of *ATG10*_rs3734114,_ we do not have enough subjects to evaluate which effect (protective or risk) prevails in case of co-occurrence, but it would be interesting to address this in the future. It would also be interesting to explore association between these two SNPs and other described SNPs impacting AML [19].

After flagging the association of *ATG10*_rs1864182G_ and *ATG10*_rs3734114C/C_ with AML, we explored which potential impact those sequence variations might have on mRNA expression and protein function in healthy individuals. Although we were able to detect alterations in the *ATG10* protein, with decreased levels of the long isoform in the presence of *ATG10*_rs1864182G_, the amount of *ATG10* transcripts was not altered. It is important to note that the primers used in our study amplify all the isoforms and do not allow a selective quantification of the different *ATG10* transcripts. A putative differential expression between transcripts could justify why *ATG10*_rs1864182_ is described to act as an expression quantitative trait locus across multiple tissues, including whole blood, in the Genotype-Tissue Expression (GTEx) project [51].

The decreased protein levels of *ATG10* long isoform in *ATG10*_rs1864182G_ carriers were associated with the reduction of autophagic flux, its canonical function, in agreement with previous observations [21]. Given the role of autophagy in leukemia [52], and in chemotherapy susceptibility and resistance [53,54,55], this reduction in autophagy could potentially be beneficial to lower the probability of developing AML. In the *ATG10* immunoblot profile (Supplementary Figure S2), we detected bands with a similar molecular weight as *ATG10* isoforms. Although not described in literature, we hypothesize that these other detected bands could correspond to yet unknown *ATG10* isoforms or even *ATG10* isoforms that underwent post-translational modifications, which may alter their mobility in the SDS-Page gel. This hypothesis should be explored further in future studies.

Regarding *ATG10*_rs3734114C/C_, due to the low number of individuals in the cohort, no conclusions can be drawn with respect to mRNA levels, protein levels and autophagy. For this reason, an increased number of homozygous mutants for *ATG10*_rs3734114_ are needed, or alternatively an in vitro approach using gene editing could be adopted.

As with *ATG10*_rs1864182_, the *ATG10*_rs3734114_ is characterized by a missense mutation resulting in a codon for a different amino acid. These mutations can potentially change the functional role of *ATG10* in autophagy and/or in other non-canonical processes in which it may be involved. Hopefully, with advances in artificial intelligence tools uncovering the 3D shapes of proteins such as AlphaFold [56], accurate predictions of protein structural changes due to SNPs will enable a better understanding of the biological implications of these mutations. Future studies should also aim to understand if any of the *ATG10* isoforms is involved in non-canonical roles [57] affecting the likelihood of AML.

## 5. Conclusions

In conclusion, we found an association between *ATG10*_rs1864182G_ and a lower-risk of developing AML, which is accompanied by an impairment of *ATG10*’s autophagy function. In addition, we describe an increased risk of developing AML in individuals carrying the *ATG10*_rs3734114C/C_. Our work reveals new mechanisms by which genetic variations in *ATG10* may coordinate the development of AML. The gathered evidence could be further exploited in prevention strategies or screening protocols of subjects carrying other risk factors for AML, such as CHIP individuals.

## Figures and Tables

**Table 1:** Demographic characterization of the two cohorts studied.

Cohort 1—Spanish Multicenter		
Healthy Donors		356
Gender	166 male	
	187 female	
	3 NA	
Age	56 ± 6 years mean age	
Acute Myeloid Leukemia		309
Gender	167 male	
	133 female	
	9 NA	
Age	58 ± 17 years mean age	
**Cohort 2—Portuguese Donors**		
Healthy Donors		46
Gender	12 male	
	34 female	
Age	39 ± 14 years mean age	

NA denotes non-available.

**Table 2:** Information regarding the *ATG10* polymorphisms. Major allele was considered the most frequent allele in the European population based on the National Library of Medicine for the corresponding SNP.

Genotyped SNPs	rs1864182	rs1864183	rs3734114
Chromosome	5	5	5
Chromosome Position	82253421	82253397	82058570
Major Allele	T	C	T
Base change	T > G	C > T	T > C
Minor Allele Frequency (MAF) in the controls	0.508	0.429	0.202
*p* value for HWE test in our controls	0.821	0.302	0.365

**Table 3:** Association of *ATG10* SNPs with Acute Myeloid Leukemia (AML). Adjusted odds ratio (OR) and 95% confidence intervals (CIs) for association between SNPs and AML were estimated using logistic regression.

SNPs	Genotypes	Donors No. (%)		χ^2^ (*p_ad_*)	LR Dominant	LR Recessive
		Control	AML		OR (CI)	OR (CI)
rs1864182	TT	77 (25)	87 (35)	0.174	0.58 (0.42–0.80) *p* = 0.001; *p_ad_ =* 0.006	0.70 (0.47–1.04) *p* = 0.077; *p_ad_ =* 0.462
	TG	154 (49)	110 (44)			
	GG	81 (26)	53 (21)			
rs1864183	CC	109 (34)	77 (29)	1.506	1.29 (0.94–1.78) *p* = 0.113; *p_ad_ =* 0.678	1.31 (0.88–1.94) *p* = 0.180; *p_ad_ =* 1.080
	CT	146 (46)	126 (47)			
	TT	63 (20)	65 (24)			
rs3734114	TT	204 (66)	168 (68)	0.012	0.86 (0.61–1.22) *p* = 0.414; *p_ad_ =* 2.484	2.70 (1.36–5.34) *p* = 0.004; *p_ad_ =* 0.024
	TC	92 (30)	53 (21)			
	CC	14 (4)	28 (11)			

Abbreviations: Logistic Regression (LR), Fisher Exact Test (FE), Chi-Square test (χ^2^), *p* value (*p*), adjusted *p* value using Bonferroni method for multiple testing (*p_ad_*).

**Table 4:** Genotype frequencies for the *ATG10*_rs1864182_ and *ATG10*_rs3734114_ in cohort 2.

SNP	Genotype	Counts (%)
rs1864182	TT	14 (30.4)
	TG	18 (39.1)
	GG	14 (30.4)
rs374114	TT	26 (56.5)
	TC	18 (39.1)
	CC	2 (4.3)

## Data Availability

The data presented in this study are available on request from the corresponding author. The data are not publicly available due to ethical reasons.

## Supplementary Materials

The following are available online at https://www.mdpi.com/2072-6694/13/6/1344/s1, Figure S1: Schematic representation of *ATG10* and the three-studied SNPs; Figure S2. The whole Western blot showing all bands and molecular weight markers of the blot represented in Figure 1D. Figure S3. The whole Western blot showing all bands and molecular weight markers of the blot represented in Figure 2A and 2H. Figure S4. The whole Western blot showing all bands and molecular weight markers of the blot represented in Figure 2O. Table S1: Association of autophagy SNPs with Acute Myeloid Leukemia (AML); Table S2: Primer sets used to perform the qPCR to evaluate *ATG10* expression (housekeepers: *GAPDH*, *RPL13A* and *B2M*).
