# Supplementary material for: Functional Genetic Variants in ATG10 Are Associated with Acute Myeloid Leukemia
Source: Cancers (Basel). 2021 Mar 16;13(6):1344. doi: 10.3390/cancers13061344 (PMC8002222; doi:10.3390/cancers13061344)
Supplement: Supplementary file 1 [file cancers-13-01344-s001.pdf]

Functional Genetic Variants in *ATG10* are Associated with Acute Myeloid Leukemia

Isabel Castro, Belém Sampaio-Marques, Anabela C. Areias, Hugo Sousa, Ângela Fernandes, José Manuel Sanchez-Maldonado, Cristina Cunha, Agostinho Carvalho, Juan Sainz and Paula Ludovico

Table S1. Association of SNPs in autophagy and other-related processes with Acute Myeloid Leukemia (AML). Adjusted odds ratio (OR) and 95% confidence intervals (CIs) for association between SNPs and AML were estimated using logistic regression. Hardy-Weinberg equilibrium for *IDH1*$_{rs11554137}$, *ATG16L1*$_{rs2241880}$, *PSMB9*$_{rs17587}$ was 0.384, 0.818, 0.871, respectively.

SNPs	Genotypes	Donors No. (%)		$\chi 2$	Dominant		Recessive		LR Dominant	LR Recessive
		Control	AML		OR (CI)	FE(p_{ad})	OR (CI)	FE(p_{ad})	OR (CI)	OR (CI)
IDH1 rs11554137	CC	272(86)	203(79)	$p = 0.050$		$p = 0.045$				
	TC	45(14)	51(20)	$p_{ad} = 0.300$	1.57 (1.01-2.44)	$p_{ad} = 0.270$	NA	NA	1.45 (0.93-2.24) $p = 0.093$ $p_{ad} = 0.558$	NA
	TT	0(0)	2(1)							
ATG16L1 rs2241880	CC	59(19)	56(23)	$p = 0.246$		$p = 0.138$		$p = 0.248$		
	CT	152(48)	124(50)	$p_{ad} = 1.476$	1.33 (0.92-1.92)	$p_{ad} = 0.828$	1.27 (0.84-1.92)	$p_{ad} = 1.488$	1.02 (0.74-1.40) $p = 0.871$ $p_{ad} = 5.226$	1.14 (0.76-1.71) $p = 0.527$ $p_{ad} = 3.162$
	TT	103(33)	66(27)							
PSMB9 rs17587	GG	186(60)	138(58)	$p = 0.808$		$p = 0.660$		$p = 0.580$		
	GA	107(35)	84(36)	$p_{ad} = 4.848$	1.08 (0.76-1.53)	$p_{ad} = 3.960$	1.23 (0.59-2.55)	$p_{ad} = 3.480$	0.96 (0.69-1.33) $p = 0.804$ $p_{ad} = 4.824$	1.25 (0.62-2.53) $p = 0.536$ $p_{ad} = 3.216$
	AA	16(5)	15(6)							
ATG10 rs1864182	TT	77(25)	87(35)	$p = 0.029$		$p = 0.009$		$p = 0.196$		
	TG	154(49)	110(44)	$p_{ad} = 0.174$	0.61 (0.42-0.88)	$p_{ad} = 0.054$	0.76 (0.51-1.13)	$p_{ad} = 1.176$	0.58 (0.42-0.80) $p = 0.001$ $p_{ad} = 0.006$	0.70 (0.47-1.04) $p = 0.077$ $p_{ad} = 0.462$
	GG	81(26)	53 (21)							
ATG10 rs1864183	CC	109(34)	77(29)	$p = 0.251$		$p = 0.155$		$p = 0.228$		
	CT	146(46)	126(47)	$p_{ad} = 1.506$	1.29 (0.91-1.83)	$p_{ad} = 0.930$	1.29 (0.87-1.91)	$p_{ad} = 1.368$	1.29 (0.94-1.78) $p = 0.113$ $p_{ad} = 0.678$	1.31 (0.88-1.94) $p = 0.180$ $p_{ad} = 1.080$
	TT	63(20)	65(24)							
ATG10 rs3734114	TT	204(66)	168(68)	$p = 0.002$		$p = 0.718$		$p = 0.003$		
	TC	92(30)	53(21)	$p_{ad} = 0.012$	0.92 (0.65-1.32)	$p_{ad} = 4.308$	2.68 (1.37-5.20)	$p_{ad} = 0.018$	0.86 (0.61-1.22) $p = 0.414$ $p_{ad} = 2.484$	2.70 (1.36-5.34) $p = 0.004$ $p_{ad} = 0.024$
	CC	14(4)	28(11)							

Abbreviations: Logistic Regression (LR), Fisher Exact Test (FE), Chi-Square test (χ^2), adjusted p value using Bonferroni method for multiple testing (p_{ad}).

Table S2. Primer sets used to perform the qPCR to evaluate *ATG10* expression (housekeepers: *GAPDH*, *RPL13A* and *B2M*).

Gene	Primer Sequence (5′-3′)	
	Forward	Reverse
ATG10	GCTTTCGAGCTACCCTTGGA	GGCACTTGGTAGCTAGAGGA
GAPDH	GTCAGTGGTGGACCTGACCT	TGACAAAGTGGTCGTTGAGG
RPL13A	CTGCCCTTCCTCCATTGTTG	CTTTCCTTGCTCCCAGCTTC
B2M	TGCTGTCTCCATGTTTGATGTATCT	TCTCTGCTCCCCACCTCTAAGT

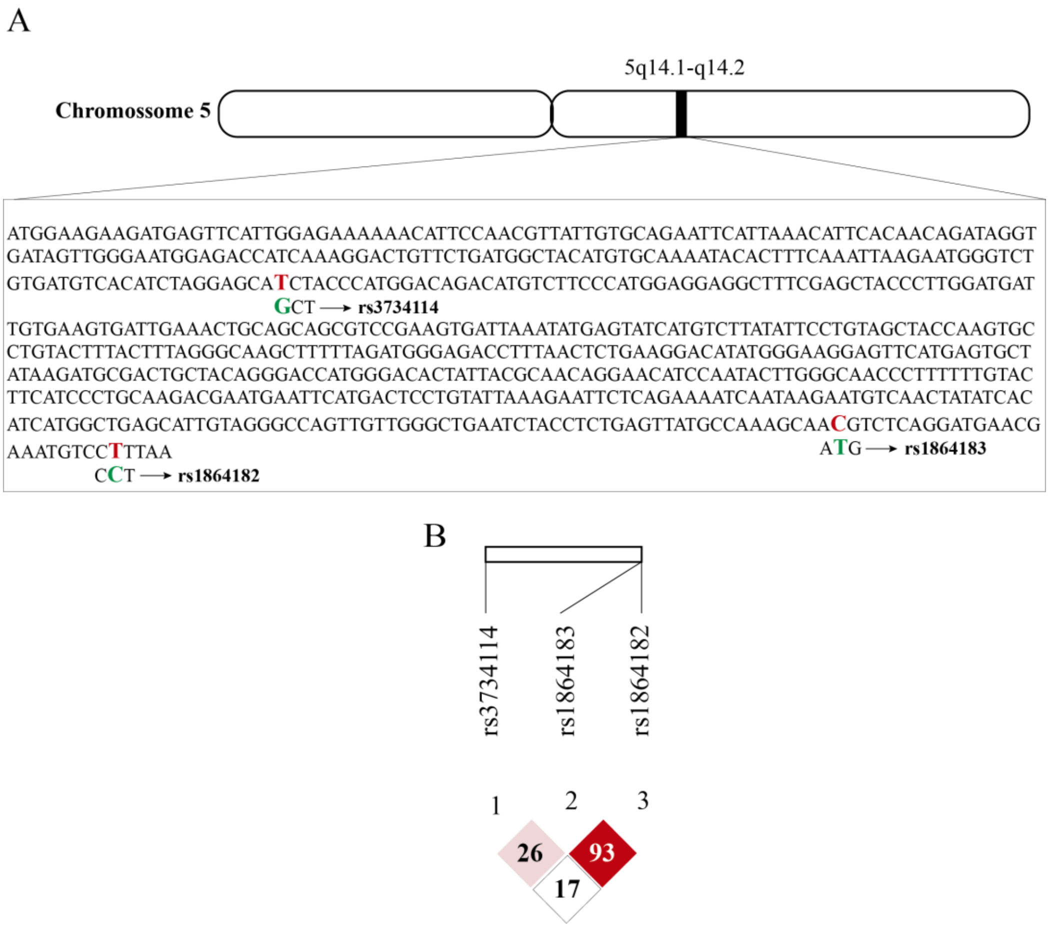

Figure S1. (**A**) Schematic representation of *ATG10* and the three-studied SNPs. *ATG10* is located at the chromosome 5 specifically at the 5q14.1-q14.2 positions. Gene sequence of *ATG10* and respective positions of $ATG10_{rs1864182}$, $ATG10_{rs1864183}$ and $ATG10_{rs3734114}$. Gene sequence retrieved from www.ncbi.nlm.nih.gov/CCDS/, acessed on 12 January 2021. (**B**) Coefficient of linkage disequilibrium (D′) for the *ATG10* SNPs. Linkage disequilibrium plot was calculated using Haploview. Represented values correspond to D′ multiplied by 100.

Figure 1D

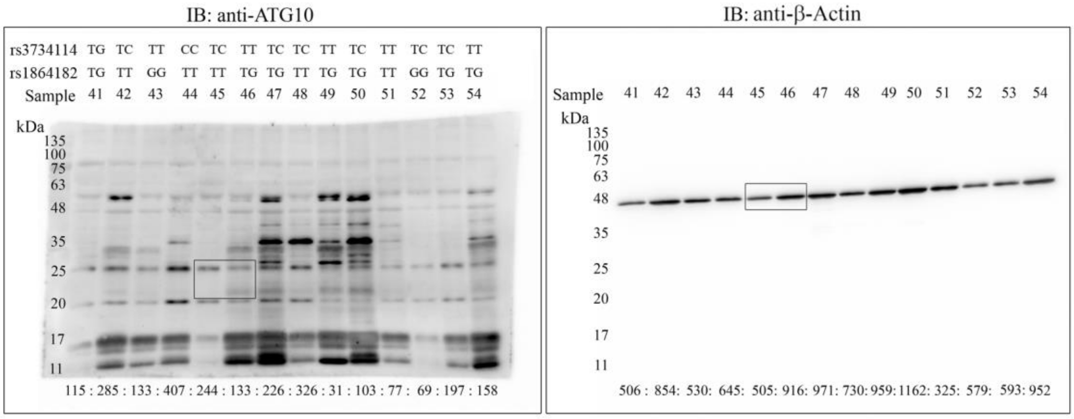

Figure S2. The whole Western blot showing all bands and molecular weight markers of the blot represented in Figure 1D.

Figure 2A and 2H

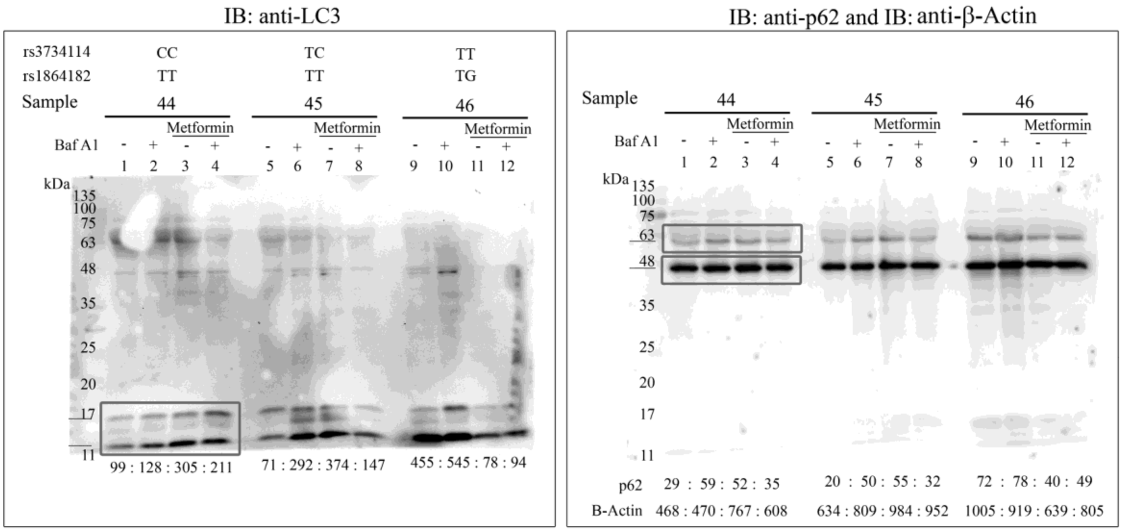

Figure S3. The whole Western blot showing all bands and molecular weight markers of the blot represented in Figure 2A and 2H.

Figure 2O

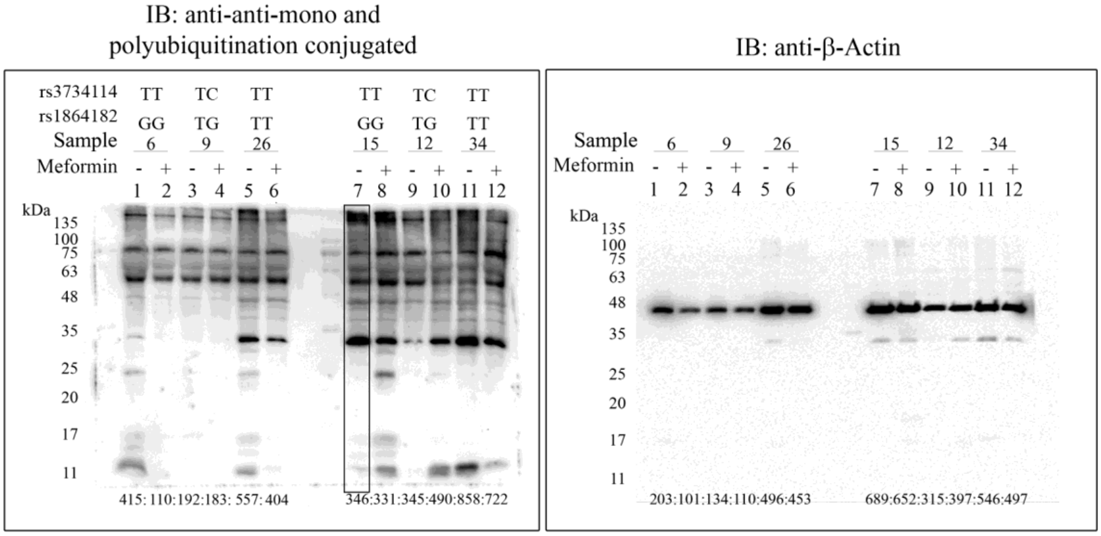

Figure S4. The whole Western blot showing all bands and molecular weight markers of the blot represented in Figure 2O.
